# Post-traumatic rectourethral fistula in an adolescent managed via a transperineal approach using a local gluteal tissue interposition flap: a case report

**DOI:** 10.1186/s40792-021-01335-z

**Published:** 2021-12-16

**Authors:** Toshinori Hirano, Hiroki Ohge, Yusuke Watadani, Shinnosuke Uegami, Norimitsu Shimada, Ikki Nakashima, Kosuke Yoshimura, Shinya Takahashi

**Affiliations:** 1 Department of Surgery, Graduate School of Biomedical and Health Sciences, Hiroshima University, 1-2-3 Kasumi, Minami-ku, Hiroshima, Hiroshima 734-8551 Japan; 2 Department of Surgery, National Hospital Organization Kure Medical Center and Chugoku Cancer Center, 3-1 Aoyama, Kure, Hiroshima 737-0023 Japan

**Keywords:** Rectourethral fistula, Adolescence, Local tissue flap

## Abstract

**Background:**

Rectourethral fistula is a rare disease with a wide variety of etiologies and clinical presentations. A definitive surgical procedure for rectourethral fistula repair has not been established.

**Case presentation:**

A 13-year-old boy sustained a penetrating injury to the perineum, and developed a symptomatic rectourethral fistula thereafter. Conservative management through urinary diversion and transanal repair was unsuccessful. Fecal diversion with loop colostomy was performed, and three months later, a fistula repair was performed via a transperineal approach with interposition of a local gluteal tissue flap. There were no postoperative complications, and magnetic resonance imaging studies confirmed the successful closure of the fistula. The urinary and fecal diversions were reverted 1 and 6 months after the fistula repair, respectively, and postoperative excretory system complications did not occur.

**Conclusions:**

The transperineal approach with interposition of a local gluteal tissue flap provides a viable surgical option for adolescent patients with rectourethral fistulas who are unresponsive to conservative management.

## Background

Rectourethral fistulas (RUFs) are abnormal communications between the rectum and urethra. They induce significant disability and have been associated with marked distress. Patients present with passage of urine from the rectum, or fecaluria, and pneumaturia. Acquired RUFs are caused by surgical complications, pelvic irradiation, malignancy, chronic infection, and trauma [1]. Conservative management includes urinary diversion (UD) with or without fecal diversion (FD) [2]. Refractory RUF cases require surgical repair [3]. Although several surgical approaches have been reported, the optimal approach has not been determined [4, 5].

## Case presentation

**Fig. 1:**
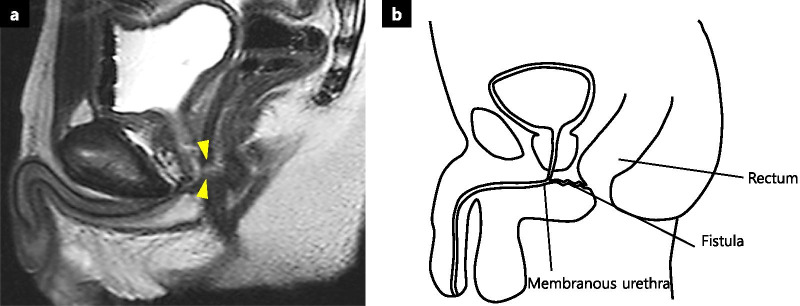
Preoperative images of rectourethral fistula. **a** Preoperative magnetic resonance imaging revealed a fistula between the posterior membranous urethra and the lower anterior rectum (yellow arrowhead). **b** Illustration of the rectourethral fistula

A 13-year-old boy sustained a penetrating injury involving the perineum. While skating, he fell and bruised his perineum with another person’s ice skate blade. He developed a RUF with pneumaturia and urine leakage through the rectum. Minimally invasive management with UD using an indwelling urinary catheter and transanal simple suture closure of the fistula failed. Six months after the injury, he was referred to the Hiroshima University Hospital. Laboratory data showed no inflammation. Pelvic magnetic resonance imaging (MRI) revealed a fistula connecting the posterior membranous urethra and the low anterior rectum (Fig. 1a, b). Three months after fecal diversion with loop colostomy, radical repair surgery was performed.

### Surgical procedure

**Fig. 2:**
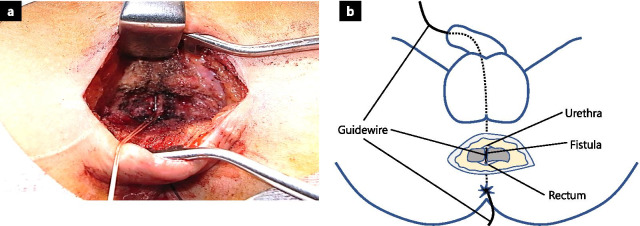
Exposure of the fistula**.** **a** The guidewire passed through the fistula from the urethral meatus to the anus. **b** Illustration of the location of fistula, organs, and guidewire

The patient was placed in a high lithotomy position following induction of general anesthesia. A guidewire (Sensor™ PTFE-Nitinol Guidewire with hydrophilic tip; Boston Scientific Corporation, Marlborough, MA, USA) was inserted using a cystoscope from the urethral meatus, passed through the fistula, and derived from the anus. A 6-cm transverse incision was made at the perineum 2-cm above the anal verge. Using electrocautery, the depth of the incision was increased in the posterior urogenital diaphragm region. Upon fistula incision, a guidewire, passing through the fistula, was encountered (Fig. 2a, b).

**Fig. 3:**
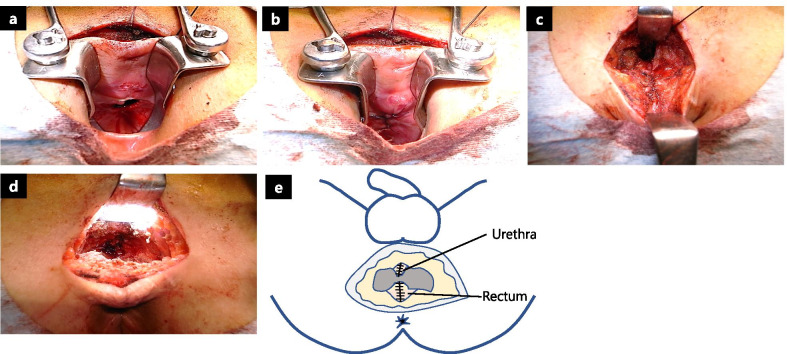
Intraoperative images. **a** Opening of the rectal mucosal surface. **b** Rectal mucosal closure via the transanal approach. **c** Adventitia of the rectum was closed from outside with absorbable sutures. **d** Urethra opening was closed with absorbable sutures. **e** Illustration of closure of the openings

**Fig. 4:**
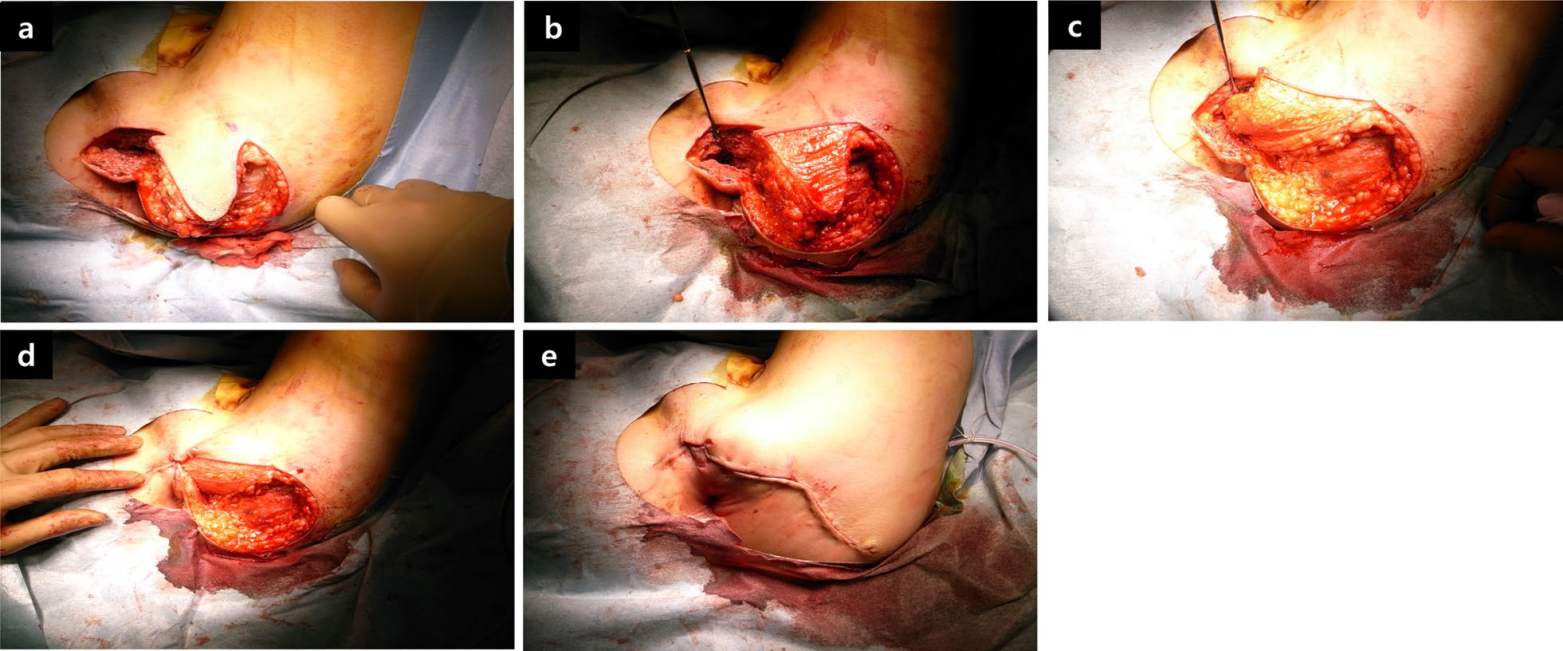
Formation and insertion of a gluteal local tissue flap. **a** Triangular design of the local tissue flap. **b** Triangular local tissue flap after its epidermis had been dissected. **c** The flap was interposed between the urethra and rectum, and fixed proximally to the fistula site. **d** The flap fits well with no deformation or bulge. **e** A closed suction drain was placed below the wound, and the surgical field closed with interrupted absorbable sutures

**Fig. 5:**
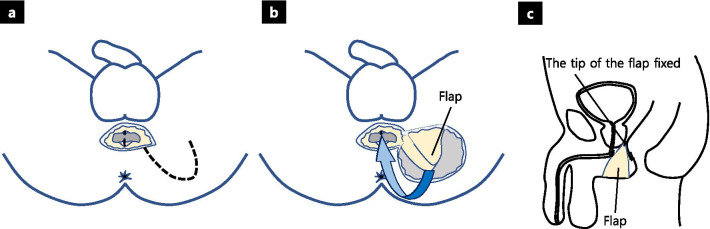
Illustrations of the operative procedure. **a** Design of the gluteal local tissue flap. **b** Rotation and insertion of the flap. **c** Sagittal section

Following complete fistula division, debridement was performed on the unhealthy and scarred tissues around the rectal and urethral openings. A simple interrupted suturing technique was performed in each layer to close the rectal and urethral openings using absorbable sutures [4-0 Vicryl; Ethicon, Johnson & Johnson, Somerville, NJ, USA (Fig. 3a–c), 4-0 PDS; Ethicon, Johnson & Johnson, Somerville, NJ, USA (Fig. 3d, e)]. A triangle-shaped local tissue flap was designed at the left lower buttock, which contains sufficiently thick and firm adipose tissue (Fig. 4a), and the epidermis of the flap was sharply incised (Fig. 4b). The flap was fully mobilized to allow rotation and then interposed between the urethra and rectum (Fig. 4c, d). A closed suction drain (Blake® silicone drain, 10Fr, round, with J-VAC® suction reservoir; Ethicon, Ethicon, Johnson & Johnson, Somerville, NJ, USA) was placed below the wound. The surgical field was irrigated and closed with interrupted absorbable sutures (4-0 Vicryl) over the flap (Figs. 4e, 5a–c). Finally, a suprapubic catheter was inserted because of the need for long-term UD. There were no complications after the surgery, and the patient was discharged 2 weeks postoperatively.

**Fig. 6:**
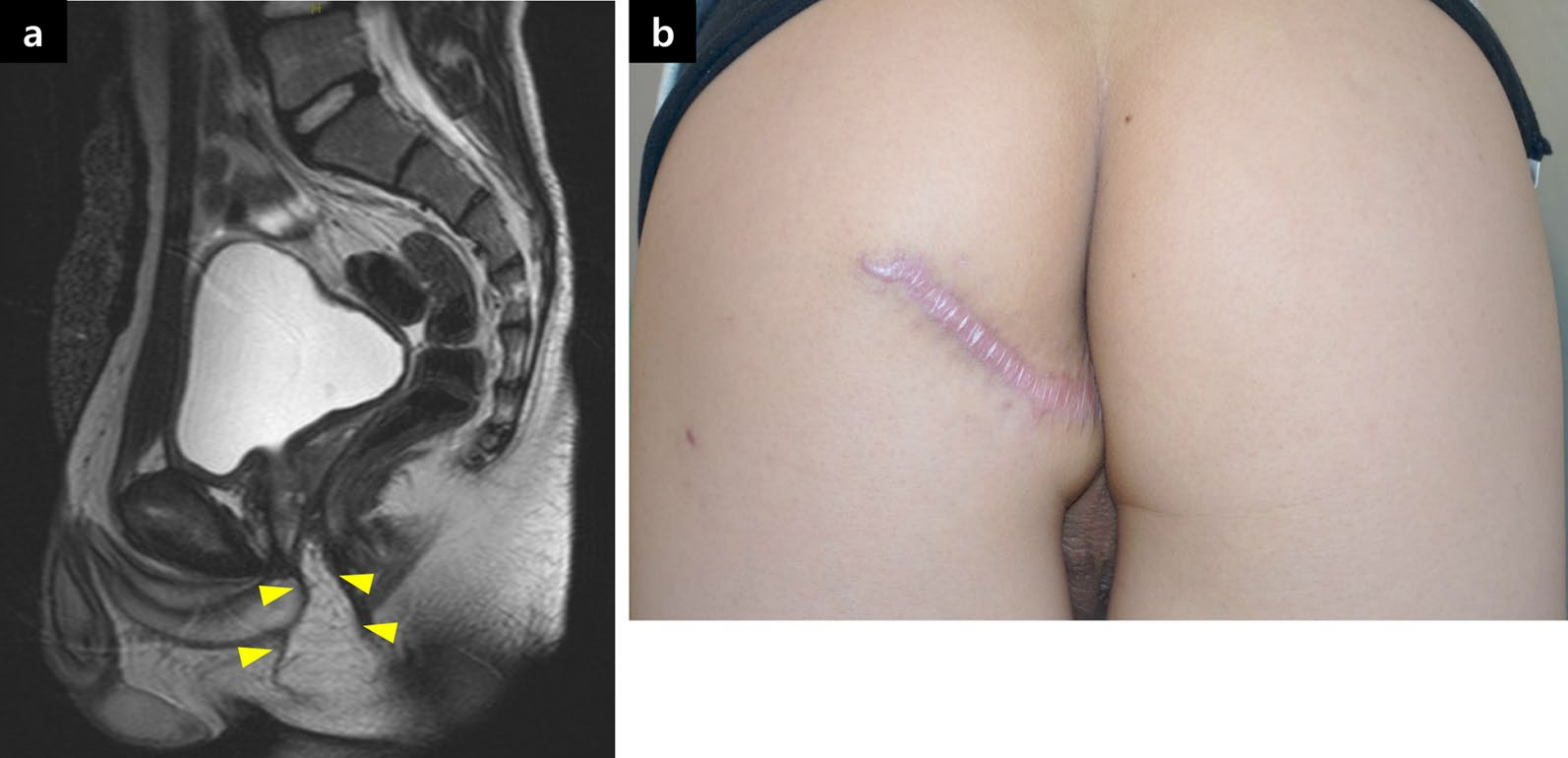
Postoperative images. **a** Pelvic magnetic resonance imaging 5 months postoperatively revealed the interposed flap between the urethra and rectum without atrophy and deviation (yellow arrowhead). **b** Scars on the buttock

One month after the procedure, urethrography showed no signs of anastomotic leakage and stricture. The suprapubic catheter was then removed. The patient had mild urinary incontinence, which improved in a few months. Additionally, urinary stream improved with no residual urine. Three months after the surgery, follow-up pelvic MRI showed a triangle-shaped local flap located between the urethra and rectum without any gaps. Two months later, a repeat MRI revealed no flap atrophy (Fig. 6a). There was also no deformity or dysfunction of the buttocks (Fig. 6b).

After confirming no RUF relapse by urethrography, the fecal diversions were reverted 6 months after the RUF repair. Postoperatively, his fecal appearance and frequency were normal with no incontinence. At present, there has been no recurrence of the RUF for more than 5 years since the surgery.

## Discussion

Acquired RUFs are commonly caused by multimodal prostate cancer treatment involving radiation [6, 7]. The causes of non-irradiated RUFs include surgery (65%), trauma (22%), and inflammatory bowel disease (6%) [5]. Conservative RUF treatment consists of initial UD with or without FD, which reportedly achieved spontaneous fistula healing in 10% of cases [5]. Surgical repair is indicated in cases where the fistula fails to heal 3 months after fecal diversion [2].

Various surgical procedures for RUF repair have been reported. The basic surgical principles are excision and debridement of the fistula tract and separation of the rectum and urethra with tissue interposition [8]. These RUF repair procedures were mainly performed via transanal, transsphincteric, transabdominal, and transperineal approaches [9]. The transperineal approach was the most commonly used, accounting for 65.9% of cases. Additionally, tissue flap interposition was done in most cases [5]. Compared to other approaches, the transperineal approach provided sufficient fistula exposure, rectal and urethral separation, and convenience in terms of flap interposition [10]. The closure rate was reportedly 91% [5].

Various tissue flaps can be interposed between the repaired urethra and rectum [11–13], but the gracilis muscle flap was utilized in over 95% of cases using the transperineal approach [5]. This muscle has been associated with a high success rate of 70–90% [14], regular blood supply, easy mobilization [10], and healthy tissue outside the irradiation field even after radiation therapy for prostate cancer. Closure failures, in cases wherein the gracilis muscle flap was used, were reportedly caused by inflammation, tissue scarring, circulatory impairment, and flap retraction due to muscle contraction [10]. According to Hampson et al., 43% of patients who underwent gracilis muscle flap reconstruction reported postoperative problems, including numbness, weakness, limited groin mobility, difficulty walking or climbing stairs, leg cramping, and leg swelling [15].

More than 80% of acquired RUF cases in children were trauma-related. Unlike adult cases, which were often related to prostate cancer therapy, the perineum and buttocks of pediatric patients were not irradiated [16]. Based on this, various tissue flaps are available, and a more appropriate flap than the gracilis muscle should be utilized to avoid complications. In our case, the transperineal approach and local gluteal tissue flap were selected after the initial transanal closure had failed. The thick and well-vascularized subcutaneous fat tissue from the lower buttock was technically easier to handle, anatomically proximal to the fistula, and provided sufficient volume to interpose the rectum and urethra without excessive invasion. Previously reported cases using local gluteal tissue flap for RUF have shown good closure rate (Table 1) [17, 18]. However, it is not indicated in patients too thin to have sufficient fatty tissue volume or with traumatic injury or infection of the buttocks, and in cases of high RUF where the gluteal local tissue flap cannot reach the fistula, due to the difficulty in forming a long flap unlike the gracilis muscle.

**Table 1:** Reported cases using local gluteal tissue flap for RUF

References	Patients, n	Etiology	Approach	Tissue flap type	Closure rate, n (%)	Temporary UD, n	Temporary FD, n
Helmy et al. [18]	1	Iatrogenic	Transperineal	Ischiorectal fat	1 (100%)	1	N/A
Levitt et al.[17]	3	N/A	N/A	Ischiorectal flap	3 (100%)	0	N/A

*N/A* not applicable, *FD* fecal diversion, *UD* urinary diversion, *RUF* rectourethral fistula

Five months postoperatively, the pelvic MRI showed no atrophy or interposed flap deviation. There were no complaints of motor or sexual dysfunction. Based on these outcomes, the gluteal local tissue flap is a viable option for young and healthy patients without a history of radiation to the perineum region.

## Conclusions

The transperineal approach with interposition of a local tissue flap from the buttocks was a viable surgical option for repairing non-irradiated RUFs in an adolescent patient that was unresponsive to conservative management.

## Data Availability

Not applicable.

## Abbreviations

FD: Fecal diversion
MRI: Magnetic resonance imaging
RUF: Rectourethral fistula
UD: Urinary diversion

## References

[1] Zmora O, Potenti FM, Wexner SD, Pikarsky AJ, Efron JE, Nogueras JJ (2003). Gracilis muscle transposition for iatrogenic rectourethral fistula. Ann Surg.

[2] Keller DS, Aboseif SR, Lesser T, Abbass MA, Tsay AT, Abbas MA (2015). Algorithm-based multidisciplinary treatment approach for rectourethral fistula. Int J Colorectal Dis.

[3] al-Ali M, Kashmoula D, Saoud IJ (1997). Experience with 30 post-traumatic rectourethral fistulas: presentation of posterior transsphincteric anterior rectal wall advancement. J Urol.

[4] Shin PR, Foley E, Steers WD (2000). Surgical management of rectourinary fistulae. J Am Coll Surg.

[5] Hechenbleikner EM, Buckley JC, Wick EC (2013). Acquired rectourethral fistulas in adults: a systematic review of surgical repair techniques and outcomes. Dis Colon Rectum.

[6] Buckley JC (2011). Complications after radical prostatectomy: anastomotic stricture and rectourethral fistula. Curr Opin Urol.

[7] Chrouser KL, Leibovich BC, Sweat SD, Larson DW, Davis BJ, Tran NV (2005). Urinary fistulas following external radiation or permanent brachytherapy for the treatment of prostate cancer. J Urol.

[8] Sotelo R, Mirandolino M, Trujillo G, Garcia A, de Andrade R, Carmona O (2007). Laparoscopic repair of rectourethral fistulas after prostate surgery. Urology.

[9] Bislenghi G, Verstraeten L, Verlinden I, Castiglione F, Debaets K, Van der Aa F (2020). Surgical management of acquired rectourethral fistula: a retrospective analysis of 52 consecutive patients. Tech Coloproctol.

[10] Nikolaev VV (2020). Recurrent rectourethral fistula repair: a novel technique of gracilis muscle interposition. J Pediatr Surg.

[11] Wexner SD, Ruiz DE, Genua J, Nogueras JJ, Weiss EG, Zmora O (2008). Gracilis muscle interposition for the treatment of rectourethral, rectovaginal, and pouch-vaginal fistulas: results in 53 patients. Ann Surg.

[12] Varma MG, Wang JY, Garcia-Aguilar J, Shelton AA, McAninch JW, Goldberg SM (2007). Dartos muscle interposition flap for the treatment of rectourethral fistulas. Dis Colon Rectum.

[13] Ganio E, Martina S, Novelli E, Sandru R, Clerico G, Realis Luc A (2013). Transperineal repair with bulbocavernosus muscle interposition for recto-urethral fistula. Colorectal Dis.

[14] Raup VT, Eswara JR, Geminiani J, Madison K, Heningburg AM, Brandes SB (2016). Gracilis muscle interposition flap repair of urinary fistulae: pelvic radiation is associated with persistent urinary incontinence and decreased quality of life. World J Urol.

[15] Hampson LA, Muncey W, Sinanan MN, Voelzke BB (2018). Outcomes and quality of life among men after anal sphincter-sparing transperineal rectourethral fistula repair. Urology.

[16] Huang X, Tan SS, Chen Y, Li T (2021). Acquired rectourethral and rectovaginal fistulas in children: a systematic review. Front Pediatr.

[17] Levitt MA, King SK, Bischoff A, Alam S, Gonzalez G, Pena A (2014). The Gonzalez hernia revisited: use of the ischiorectal fat pad to aid in the repair of rectovaginal and rectourethral fistulae. J Pediatr Surg.

[18] Helmy TE, Sarhan OM, Dawaba ME, Wadie BS (2010). Urethrorectal fistula repair in children: urologic perspective. J Trauma.

